# Membrane coordination of receptors and channels mediating the inhibition of neuronal ion currents by ADP

**DOI:** 10.1007/s11302-016-9516-5

**Published:** 2016-05-12

**Authors:** Hend Gafar, Manuel Dominguez Rodriguez, Giri K. Chandaka, Isabella Salzer, Stefan Boehm, Klaus Schicker

**Affiliations:** Department of Neurophysiology and Neuropharmacology, Center for Physiology and Pharmacology, Medical University of Vienna, Schwarzspanierstrasse 17/I, A-1090 Vienna, Austria

**Keywords:** P2Y receptors, Calcium channel, Kv7 channel, FRET, TIRF

## Abstract

ADP and other nucleotides control ion currents in the nervous system via various P2Y receptors. In this respect, Cav2 and Kv7 channels have been investigated most frequently. The fine tuning of neuronal ion channel gating via G protein coupled receptors frequently relies on the formation of higher order protein complexes that are organized by scaffolding proteins and harbor receptors and channels together with interposed signaling components. However, ion channel complexes containing P2Y receptors have not been described. Therefore, the regulation of Cav2.2 and Kv7.2/7.3 channels via P2Y1 and P2Y12 receptors and the coordination of these ion channels and receptors in the plasma membranes of tsA 201 cells have been investigated here. ADP inhibited currents through Cav2.2 channels via both P2Y1 and P2Y12 receptors with phospholipase C and pertussis toxin-sensitive G proteins being involved, respectively. The nucleotide controlled the gating of Kv7 channels only via P2Y1 and phospholipase C. In fluorescence energy transfer assays using conventional as well as total internal reflection (TIRF) microscopy, both P2Y1 and P2Y12 receptors were found juxtaposed to Cav2.2 channels, but only P2Y1, and not P2Y12, was in close proximity to Kv7 channels. Using fluorescence recovery after photobleaching in TIRF microscopy, evidence for a physical interaction was obtained for the pair P2Y12/Cav2.2, but not for any other receptor/channel combination. These results reveal a membrane juxtaposition of P2Y receptors and ion channels in parallel with the control of neuronal ion currents by ADP. This juxtaposition may even result in apparent physical interactions between receptors and channels.

## Introduction

P2Y receptors are a group of G protein coupled receptors (GPCRs) that are expressed in almost every tissue of the vertebrate body, including the nervous system [1]. They are activated by nucleotides such as ATP, ADP, UTP, or UDP. To date, eight different subtypes are known which are preferentially linked to either Gαq (P2Y1, P2Y2, P2Y4, P2Y6, P2Y11) or Gαi (P2Y12, P2Y13, and P2Y14) subunits of heterotrimeric G proteins [2]. Many of these receptors have been shown to regulate the functions of neuronal ion channels [3]. For example, the ADP sensitive P2Y1 receptor mediates inhibition of Kv7 potassium channels [4], K2P channels [5], Cav2.2 calcium channels [6], and NMDA glutamate receptors [7] as well as opening of KCa2 potassium channels [8, 9]. Similarly, activation of P2Y2 receptors leads to gating of KCa3 [10, 11], CFTR [12, 13], as well as calcium-activated chloride [13] channels and to a reduction of NMDA receptor currents [14]. P2Y12 receptors are involved in the inhibition of calcium channels [15, 16] and K2P channels [5] by ADP.

According to the fluid mosaic model of the membrane, proteins embedded therein may diffuse in a two-dimensional manner and randomly collide with each other [17]. This also holds true for GPCRs which can diffuse in the membrane and activate the G proteins through collision coupling [18]. However, from cell-attached patch-clamp recordings, it is known that the modulation of ion channels via either Gαi or Gαq coupled receptors is confined to a rather limited region surrounding the channels: in these experiments, ion channels enclosed by a patch pipette were not regulated if the appropriate agonists were applied to membrane regions not covered by the pipette, but only when they were present in the pipette solution [19, 20]. These findings gave rise to the idea that channels and GPCRs have to be in close proximity for the latter to exert their modulatory actions on the channel. From beta adrenergic receptors and metabotropic glutamate receptors, it is well known that scaffolding proteins like PSD95 or NHERF1/2 tether these to their effectors like NMDA glutamate receptors or CFTR [21]. In accordance with this notion, P2Y1 receptors were found to interact with NHERF2, and this interaction enhanced the signaling via Gαq [22]. In sympathetic neurons, however, which are usually devoid of NHERF2, expression of this protein attenuated the inhibition of calcium, but not that of Kv7 potassium channels via P2Y1 [23]. The closure of Kv7 channels in these neurons via P2Y6 receptors [24] is also independent of yet another scaffolding protein, AKAP79 [25]. Similarly, P2Y12 receptors do interact with NHERF1, but this is not required for signaling via Gαi [26]. Taken together, evidence for scaffold protein interactions of P2Y receptors as prerequisite for their signaling is rather limited and has not been demonstrated for the control of ion channels.

Nevertheless, the tight regulation of ion channels via P2Y receptors has been observed repeatedly in neurons [3] and has even been reconstituted in non-neuronal cells by heterologous coexpression of, for instance, P2Y12 receptors and Kir3.1/3.2 concatemers [27] or P2Y2 receptors and Kv7 channels [28]. Likewise, endogenous P2Y13 receptors of HEK293 cells mediate an inhibition of recombinant Cav2.2 channels [29]. At least for Kir3.1 and Kir3.2, direct interactions with activating GPCRs have been demonstrated [30, 31]. P2Y receptors have a tendency to interact with a number of different membrane proteins including other GPCRs and enzymes as shown by FRET measurements [32]. Hence, it was tempting to speculate that P2Y receptors might also get in contact with neuronal ion channels, in particular Cav2 and Kv7 family members, as basis for the regulation of neuronal ion currents by nucleotides. Accordingly, electrophysiological and imaging methods were employed here to reveal whether direct interactions between P2Y1 and P2Y12 receptors, on one hand, and Cav2.2 and Kv7.2/Kv7.3 channels, on the other hand, do occur in parallel to the modulation of neuronal ion currents by ADP.

## Methods

### Cell culture and transfection

The cell line tsA 201, a subclone of HEK293 cells stably expressing the SV40 large T-antigen, was cultured in antibiotic free Dulbecco’s modified Eagle’s medium (DMEM; Sigma-Aldrich, Vienna, Austria) supplemented with 10 % fetal bovine serum (Invitrogen, Lofer, Austria). For patch-clamp experiments, cells were seeded onto conventional 35 mm culture dishes (Nunc, Roskilde, Denmark), whereas for fluorescence microscopy the transfected cells were plated on poly-D-lysine-coated glass cover slips or glass bottom dishes (IBIDI, Martinsried Germany). For the transfection of calcium channels into tsA 201 cells prior to electrophysiological recordings, JetPrime® (Polyplus Transfection, Illkirch, France) was used according to the manufacturer’s recommendations. For all other transfections, the Turbofect® reagent (Thermo Fischer Scientific, Vienna, Austria) was used as follows: per 35 mm dish, a total of 1 μg DNA was mixed with 200 μl of 150 mM NaCl and 5 μl of transfection reagent, and after 10–15 min preincubation, this mixture was added to the cell cultures.

Most of the plasmids as used here have been described before in more detail (Schicker et al. 2009): P2X2-CFP/-YFP, P2Y1-CFP/-YFP, P2Y12-CFP/-YFP, and NTPDase1-YFP. Plasmids for fluorescently tagged human Kv7.2 and rat Kv7.3 as well as human M1 muscarinic receptors were provided by Mark Shapiro (San Antonio, TX, USA). A construct coding for human Cav2.2-GFP was obtained from Gerald Obermair (Innsbruck, Austria), and the GFP tag was exchanged for EYFP. Cav2.2 was always coexpressed together with accessory rat β3 and rabbit α2δ1 subunits (from G. Obermair). The identities of all plasmids were verified by sequence analysis. When non-fluorescent proteins were expressed, EGFP was used to identify successfully transfected cells.

### Electrophysiology

Patch pipettes were fabricated from borosilicate glass capillaries (GB150-8P, Science Products, Hofheim, Germany) with a Sutter P97 puller (Sutter Instruments, Novato, CA, USA). Electrodes had tip resistances between 2 and 5 MΩ when filled with recording solution (see below). After establishing the recording conditions, series resistance values were between 10 and 20 MΩ and were routinely compensated for by 60–70 %. Electrophysiological recordings were carried out at room temperature (20–24 °C) from tsA 201 cells approximately 48 h after transfection. Currents through Cav2.2 channels were recorded in whole-cell mode with a pipette solution consisting of the following (in mM): CsOH (145), L-aspartic acid (145), MgCl_2_ (2), HEPES (10), Cs-EGTA (0.1), and Mg-ATP (2), adjusted to pH 7.4 with Cs-OH. For Ca^2+^ currents, the external bath solution consisted of the following (in mM): CaCl_2_ (10), TEA-Cl (145), and HEPES (10), adjusted to pH 7.4 with TEA-OH. With these solutions, the liquid junction potential is close to zero. Ca^2+^ currents were elicited once every 15 s by 30 ms depolarizations from a holding potential of −80 to +20 mV. The currents were quantified by measuring peak current amplitudes unless indicated otherwise.

Currents through the Kv7.2/7.3 channels were recorded in perforated-patch mode to minimize their rundown. Pipettes were first front-filled with a solution containing (mM): KGluconate (133), NaCl (5.9), CaCl_2_ (1), MgCl_2_ (0.7), HEPES (10), EGTA (10) adjusted to pH 7.4 with KOH. Thereafter, the electrodes were backfilled with the same solution containing 200 μg/ml amphotericin B (in 0.8 % DMSO). In these experiments, the external bath solution consisted of (mM): NaCl (140), CaCl_2_ (2.5), MgCl_2_ (2), KOH (3), glucose (20) and HEPES (10), adjusted to pH 7.4 with NaOH. These solutions result in a liquid junction potential of about 15 mV which was corrected for during experimentation. To activate these currents, cells were held at a potential of −30 mV, and once every 15 s currents were deactivated by 1 s hyperpolarizations to −55 mV. Currents through Kv7 channels were quantified by determination of deactivation amplitudes as described before [24].

### Epifluorescence microscopy

Epifluorescence three filter Förster resonance energy transfer (FRET) experiments were performed as described previously [32]. In brief, tsA 201 cells were transfected as described above. Twenty-four hours after transfection, cells were subjected to experiments at an inverted microscope using a ×63 NA 1.4 oil immersion objective (Zeiss, Vienna, Austria). Immediately before the experiment, the culture medium was exchanged by a buffer containing the following (in mM): NaCl (140), CaCl_2_ (2.5), MgCl_2_ (2), KOH (3), glucose (20), and HEPES (10), adjusted to pH 7.4 with NaOH. Fluorescent proteins were exited using the emission of a 100-W mercury burner (Zeiss) passed through a dielectric filter (436/20 ECFP, 500/20 EYFP; Chroma, CT, USA) mounted in a filter wheel (Ludl, NY, USA) allowing for fast change of excitation filters. Resulting fluorescence was passed through a fixed double band dichroic mirror (Chroma, CT, USA) and a dielectric emission filter (480/40 ECFP, 535/30 EYFP/FRET), again mounted in a filter wheel. Images were captured using a cooled CCD camera (Coolsnap fx, Roper Scientific). For measurement of FRET, three images of each cell were captured: one donor (donor excitation/donor emission, *I*_D_), one acceptor (acceptor excitation/acceptor emission, *I*_A_), and one FRET image (donor excitation/acceptor emission, *I*_DA_). Normalized FRET (NFRET) was calculated according to the formula NFRET = (*I*_DA_ − *α*_ID_ − *β*_IA_) / sqrt(*I*_D_ × *I*_A_), with *α* and *β* being spectral bleedthrough factors measured in cells expressing donor or acceptor only [33].

### Confocal microscopy

To visualize membrane expression of receptors and ion channels, tsA 201 cells were transfected as described above. Images were captured on a Zeiss LSM510 using a ×63 NA 1.4 objective employing the 458 nm (ECFP) and 514 nm (EYFP) laser line of an argon gas laser. The pinhole was set to give a 1-μm optical section. To evaluate colocalization, images were background corrected, intensities for each channel were normalized, and channels were overlaid thereafter.

### TIRF microscopy

To visualize fluorescence emission from CFP or YFP-tagged proteins in the immediate vicinity of the membrane, total internal reflection microscopy (TIRF) was employed [34]. All TIRF experiments were performed on an iMIC Digital TIRF microscope (FEI, Munich, Germany) using a ×60 NA 1.49 Objective (Olympus, Vienna, Austria) in 360° rotating TIRF mode [35]. Images were captured using a cooled EMCCD camera (Andor, Belfast, UK). TIRF angles were calibrated for each sample using the built in calibration routine. All measurements were conducted using the same nominal calculated penetration depth (80 nm).

Donor recovery after acceptor photobleaching (DRAP) was used to measure FRET efficiency directly in TIRF mode. To this end, tsA 201 cells on poly-D-lysine-coated glass bottom dishes were used 48 h after transfection. Immediately before the experiment, the culture medium was exchanged by a buffer containing the following (in mM): NaCl (140), CaCl_2_ (2.5), MgCl_2_ (2), KOH (3), glucose (20), and HEPES (10), adjusted to pH 7.4 with NaOH. For measuring FRET efficiency images of both, ECFP and EYFP images were captured, followed by a 240-s bleaching step of the entire field of view using a 150-W Xenon lamp (FEI, Munich) passed through an EYFP excitation filter (Chroma, USA). This reduced the intensity of EYFP by 97.9 ± 0.4 %. Images were analyzed using ImageJ (NIH, MD, USA) and FRET efficiency was calculated as the percentage increase in CFP emission after YFP photobleaching [25].

For fluorescence recovery after photobleaching (FRAP), a region of interest (ROI) was selected and five images were taken before and up to 100 after bleaching, both in TIRF mode at a frequency of 0.5 Hz. For bleaching, the illumination was switched to epifluorescence mode, and the preselected ROI was bleached using the 488-nm laser line. Analysis was performed using ImageJ. After subtracting the background, the fluorescence intensity of the bleached ROI was measured at each time point. To correct for bleaching caused by image acquisition, a control ROI in an adjacent, non-bleached cell was followed for its fluorescence intensity over time. All values were normalized to the mean value of the five pre-bleach images and afterwards corrected for acquisition-induced bleaching according to the following formula: FRAP_corr_ = (FRAP_norm_ + (1 − CTRL_norm_)).

## Results

To investigate whether the modulation of neuronal ion currents by ADP is paralleled by a certain spatial arrangement of P2Y receptors and ion channels, P2Y1 and P2Y12 receptors, respectively, were expressed in tsA 201 cells together with either Kv7.2/Kv7.3 heteromers or Cav2.2 channels. First, electrophysiological experiments were carried out to reveal which of these two ion channels can be controlled via either of these two ADP receptors. Thereafter, the spatial relation between receptors and channels was investigated using FRET and a potential physical interaction was tested for via FRAP.

### ADP controls the gating of Kv7 channels via P2Y1, but not via P2Y12 receptors

**Fig. 1:**
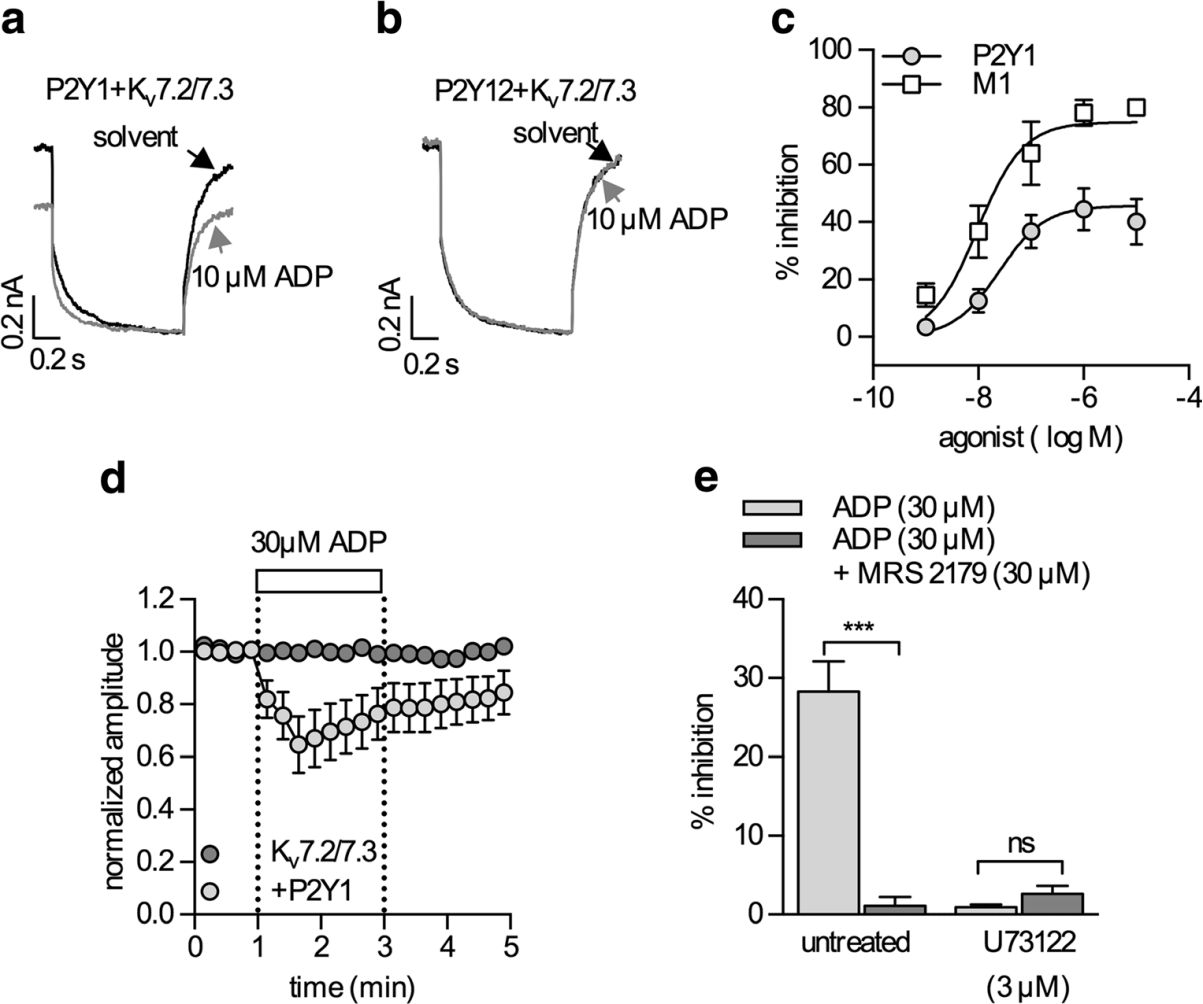
Modulation of Kv7 channels via P2Y receptors. Current responses were recorded from tsA 201 cells expressing Kv7.2/7.3 heteromers together with P2Y1 (**a**, **c**, **d**), P2Y12 (**b**), or M1 muscarinic (**c**) receptors. Cells were clamped to a voltage of −30 mV and hyperpolarized to −55 mV for 1 s once every 15 s. **a**, **b** Original current traces obtained in the presence of either solvent (*black*) or ADP (*gray*). **c** Concentration response relation for the inhibitory action of ADP (*gray circles*) and the M1 receptor agonist oxotremorine M (*white squares*) on currents through Kv7.2/7.3 heteromers (*n* = 5). **d** Time course of normalized current amplitudes in cells expressing Kv7.2/7.3 alone (*dark gray*; *n* = 6) or Kv7.2/7.3 together with P2Y1 receptors (*light gray*; *n* = 6); ADP was present as indicated by the bar. **e** Effects of ADP and MRS2197 in either untreated cells (*n* = 8) or cells pretreated with 3 μM U73122 for 8 min (*n* = 6). *Triple asterisks* (***) indicate *p* < 0.001, *ns* indicates no significant difference

In cells that had not been transfected with plasmids coding for P2Y receptors, currents through heteromeric Kv7.2/7.3 channels were not altered in the presence of 100 μM ADP (99.7 ± 0.1 % of control; *n* = 6). Likewise, in cells coexpressing P2Y12 receptors, this ADP concentration failed to cause alterations in current amplitudes (99.6 ± 0.3 % of control; *n* = 6; Fig. 1b). In contrast, in cells coexpressing P2Y1 receptors, ADP reduced current amplitudes in a concentration-dependent manner with an EC_50_ of 25 nM (95 % confidence interval, 14.7–42.5 nM) and maximal inhibition by 45.7 ± 7.2 % (Fig. 1a, c). For comparison, activation of coexpressed M1 muscarinic receptors by increasing concentrations of oxotremorine M reduced currents through Kv7.2/7.3 channels by up to 76.3 ± 6.4 % with an EC_50_ of 9.2 nM (95 % confidence interval, 4.0–16.9 nM; Fig. 1c). The inhibition of Kv7 channels via P2Y1 receptors reached a maximum within <1 min and displayed some decline thereafter despite the continuing presence of ADP (Fig. 1d). This inhibitory action of ADP was abolished by the specific P2Y1 receptor antagonist MRS2179 (30 μM; Fig. 1e).

To gain insight into the underlying signaling mechanisms, cells coexpressing P2Y1 receptors and Kv7.2/7.3 heteromers were incubated in the phospholipase C inhibitor U73122 (3 μM) for 8 min and were then exposed to 30 μM ADP, either alone or in combination with MRS 2179 (30 μM; Fig. 1d). Under these conditions, ADP did not reduce current amplitudes, and the results obtained with and without MRS 2179 were the same (Fig. 1e).

### ADP controls the gating of Cav2.2 channels via P2Y1 and P2Y12 receptors

**Fig. 2:**
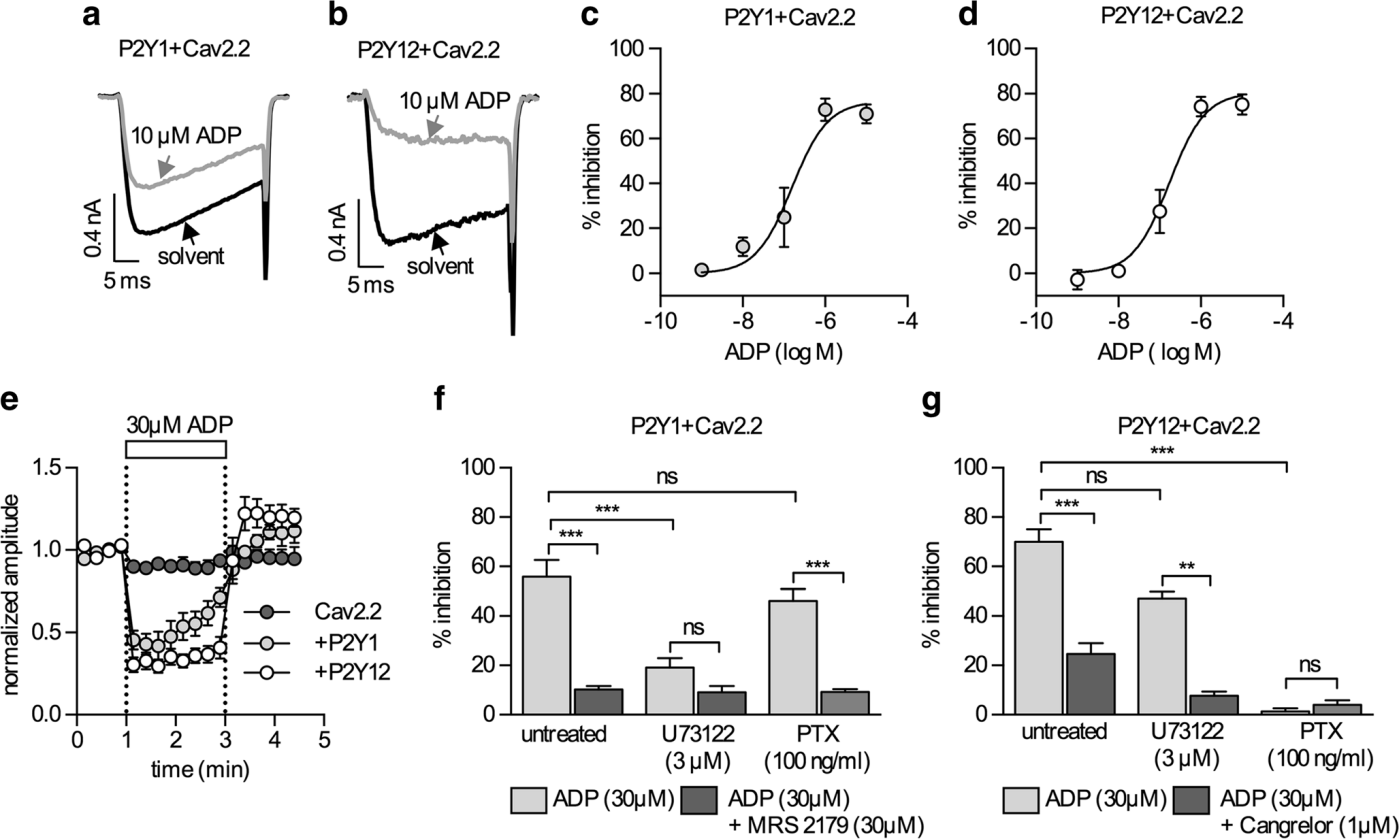
Modulation of Cav2.2 channels via P2Y receptors. Current responses were recorded from tsA201 cells expressing Cav2.2 (plus β3 and α2δ) together with either P2Y1 or P2Y12 receptors. Cells were clamped to a voltage of −80 mV and depolarized to +20 mV for 30 ms once every 15 s. **a**, **b** Original current traces obtained in the presence of either solvent (*black*) or ADP (*gray*). **c**, **d** Concentration response relations for the inhibitory actions of ADP on Ca^2+^ currents in cells expressing P2Y1 (**c**) or P2Y12 (**d**) receptors (*n* = 5 in both graphs). **e** Time course of normalized current amplitudes in cells expressing Cav2.2 alone (*dark gray*; *n* = 12), Cav2.2 together with P2Y1 receptors (*light gray*; *n* = 12), or Cav2.2 together with P2Y12 receptors (*white*; *n* = 14); ADP was present as indicated by the bar. **f** Effects of ADP and MRS2197 in cells expressing Cav2.2 together with P2Y1 receptors which were either untreated (*n* = 9), pretreated with 3 μM U73122 for 8 min (*n* = 9), or pretreated with PTX (100 ng/ml) for 24 h (*n* = 9). **f** Effects of ADP and cangrelor in cells expressing Cav2.2 together with P2Y12 receptors which were either untreated (*n* = 9), pretreated with 3 μM U73122 for 8 min (*n* = 9), or pretreated with PTX (100 ng/ml) for 24 h (*n* = 9). *Double asterisks* (**), *triple asterisks* (***) indicate *p* < 0.01 and *p* < 0.001, respectively; *ns* indicates no significant difference

Currents through Cav2.2 channels elicited by a 30-ms depolarization to 20 mV were not influenced by ADP (30 μM) in cells that had not been transfected with P2Y receptor plasmids, and current amplitudes in the presence of the nucleotide amounted to 99.7 ± 0.1 % of control (*n* = 8). However, when either P2Y1 (Fig. 2a, c) or P2Y12 (Fig. 2b, d) receptors were coexpressed with the Ca^2+^ channels, ADP clearly reduced current amplitudes in a concentration-dependent manner; the according EC_50_ values were 153 nM (95 % confidence interval, 64.7–359.4 nM) for P2Y1 and 165 nM (95 % confidence interval, 89.2–305.4 nM) for P2Y12. In both cases, the maximal inhibition was about 80 %. Nevertheless, the inhibition via these two types of P2Y receptor was not identical: activation of P2Y1 receptors reduced current amplitudes to the same extent after 5 (42.5 ± 5.2 % of control) and 25 ms (41.7 ± 5.8 % of control; *n* = 13; *p* > 0.7) of the depolarization, whereas activation of P2Y12 caused significantly more inhibition after 5 (35.0 ± 6.8 % of control) than after 25 ms (40.2 ± 8.2 % of control; *n* = 12; *p* < 0.05). This latter difference reflects a slowing of channel gating and is characteristic of the voltage-dependent inhibition of Cav2 channels via G protein βγ subunits [36–38]. Another difference between these two types of inhibition became obvious when comparing the time course: current suppression via P2Y1 receptors faded while ADP (30 μM) was present, whereas current inhibition by the nucleotide remained stable over 2 min when mediated by P2Y12 (Fig. 2e).

In cells expressing P2Y1 receptors, Ca^2+^ current reduction by ADP was antagonized by MRS2179 (30 μM; Fig. 2f). In the case of P2Y12 receptor coexpression, the inhibition was attenuated by cangrelor (1 μM; Fig. 2g), a specific P2Y12 receptor blocker. Similar to these antagonists, interference with the underlying signaling cascades was also able to differentiate between the two receptors. The inhibition via P2Y1 receptors was attenuated when the cells had been incubated in the phospholipase C inhibitor U73122 (3 μM for 8 min), but was not altered subsequent to an incubation in pertussis toxin (100 ng/ml for 24 h). Exactly the opposite was true in the case of P2Y12 receptors. When these underlying signaling cascades had been blocked, the appropriate antagonists did not alter the action of ADP anymore (Fig. 2f, g).

### Membrane juxtaposition of P2Y receptors and ion channels

**Fig. 3:**
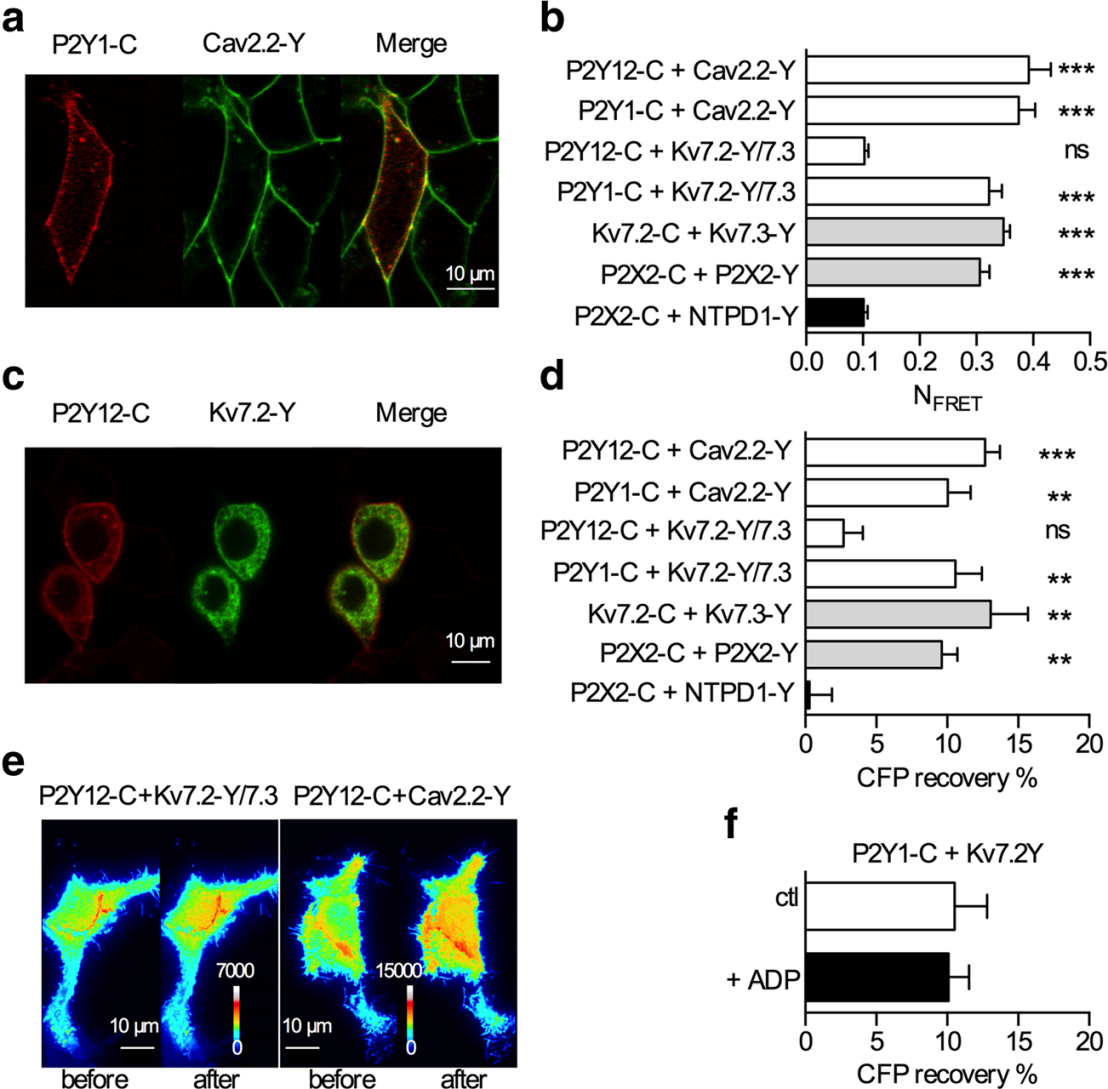
Juxtaposition of P2Y receptors and ion channels assayed by FRET microscopy. **a** Confocal images of tsA 201 cells expressing CFP-tagged P2Y1 receptors (*left*) and YFP-tagged Cav2.2 channels (*middle*) and the channel overlay (*right*). **b** *N*_FRET_ values obtained with the fusion proteins mentioned above, with P2Y1, Kv7.2, and P2X2 fused to CFP, and with Kv7.3, P2X2, and NTPDase1 (NTPD1) fused to YFP. The values obtained with P2X2-C plus P2X2-Y and P2X2-C plus NTPD1-Y were used as positive and negative controls, respectively (*n* = 20). **c** Confocal images of tsA 201 cells expressing CFP-tagged P2Y12 receptors (*left*) and YFP-tagged Kv7.2 channels (*middle*) and the channel overlay (*right*). **d** Shown is the increase in CFP emission after YFP photobleaching (*n* = 12). *Double asterisks* (**) and *triple asterisks* (***) indicate significant differences versus the values obtained with P2X2-C plus NTPD1-Y at *p* < 0.01 and *p* < 0.001, respectively; *ns* indicates no significant difference. **e** Shown are tsA 201 cells under TIRF illumination expressing CFP-tagged P2Y12 receptors and YFP-tagged Kv7 or Cav2.2 channels. Images of CFP emission (in rainbow pseudocolor) are shown before or after YFP photobleaching, as indicated. **f** Increase in CFP emission after YFP photobleaching in cells expressing P2Y1-CFP and Kv7.2-YFP in the absence (*white bar*) and presence (*black bar*) of 10 μM ADP (*n* = 5)

Confocal images of tsA 201 cells expressing either fluorescently labeled P2Y1 or P2Y12 receptors together with analogously tagged Kv7 or Cav2.2 channels revealed colocalization of both types of proteins at the plasma membrane. Although this colocalization was better visible for Cav2.2 than for Kv7 channels (Fig. 3a, c), this does not necessarily coincide with either loose or tight apposition of the investigated proteins [32]. To obtain insight into the adjacency of receptors and ion channels, FRET measurements were performed, first via epifluorescence microscopy. Using fluorescently labeled receptors and calculations of *N*_FRET_ values, we have shown that P2Y1 and P2Y12 receptors can form homomers and heteromers with other GPCRs and with NTPDases [32]. The same technique was employed here to reveal whether these receptors can be found in contiguity with the ion channels they regulate. As reported previously [32], *N*_FRET_ values obtained with ECFP- and EYFP-labeled P2X2 subunits served as positive controls, whereas those retrieved with P2X2-ECFP and NTPDase1-EYFP were used as negative controls. To confirm these controls, EYFP-tagged Kv7.2 was coexpressed with ECFP-tagged Kv7.3, as these ion channel subunits are known to form heteromers and to produce FRET [39]. The *N*_FRET_ values obtained with these Kv7 channel subunits were indiscernible from those seen with ECFP- and EYFP-labeled P2X2 subunits and significantly different from the negative controls (Fig. 3b). In this experimental paradigm, epifluorescence measurements revealed that both, P2Y1 and P2Y12, each carrying a C-terminal ECFP, can be found straightly adjacent to EYFP-tagged Cav2.2 channels, whereas only P2Y1 but not P2Y12 can be found in juxtaposition with Kv7.2/7.3 heteromers in which Kv7.2 carried the EYFP tag (Fig. 3b).

In epifluorescence FRET measurements, a significant proportion of the signal might be derived rather from membrane-associated intracellular compartments than from the plasma membrane itself. Therefore, FRET measurements were repeated employing TIRF microscopy together with DRAP to obtain results directly derived from the plasma membrane. As positive controls, Kv7.2-EYFP together with Kv7.3-ECFP and P2X2-ECFP together with P2X2-EYFP were employed, and P2X2-ECFP plus NTPDase1-EYFP served as negative control again. In the latter case, no fluorescence recovery was observed, whereas the recovery amounted to about 10 % with P2X2 homomers and Kv7.2/7.3 heteromers (Fig. 3d). With Cav2.2 channels, the DRAP values obtained for both P2Y receptors were comparable to those of the P2X2 and Kv7 subunits and significantly different from the negative control. With Kv7.2-YFP/7.3 heteromers, only the results for P2Y1-ECFP, but not those for P2Y12-ECFP, were significantly different from the negative controls (Fig. 3b, d). These data indicate that only those ADP receptors that mediate a modulation of currents reside in the membrane in juxtaposition to the respective ion channels.

To reveal whether this juxtaposition might change upon receptor activation, DRAP experiments were repeated in cells coexpressing Kv7 channels and P2Y1 receptors in the absence as well as presence of ADP (10 μM). However, the nucleotide did not cause any change (Fig. 3f).

### Physical interactions between P2Y receptors and ion channels

**Fig. 4:**
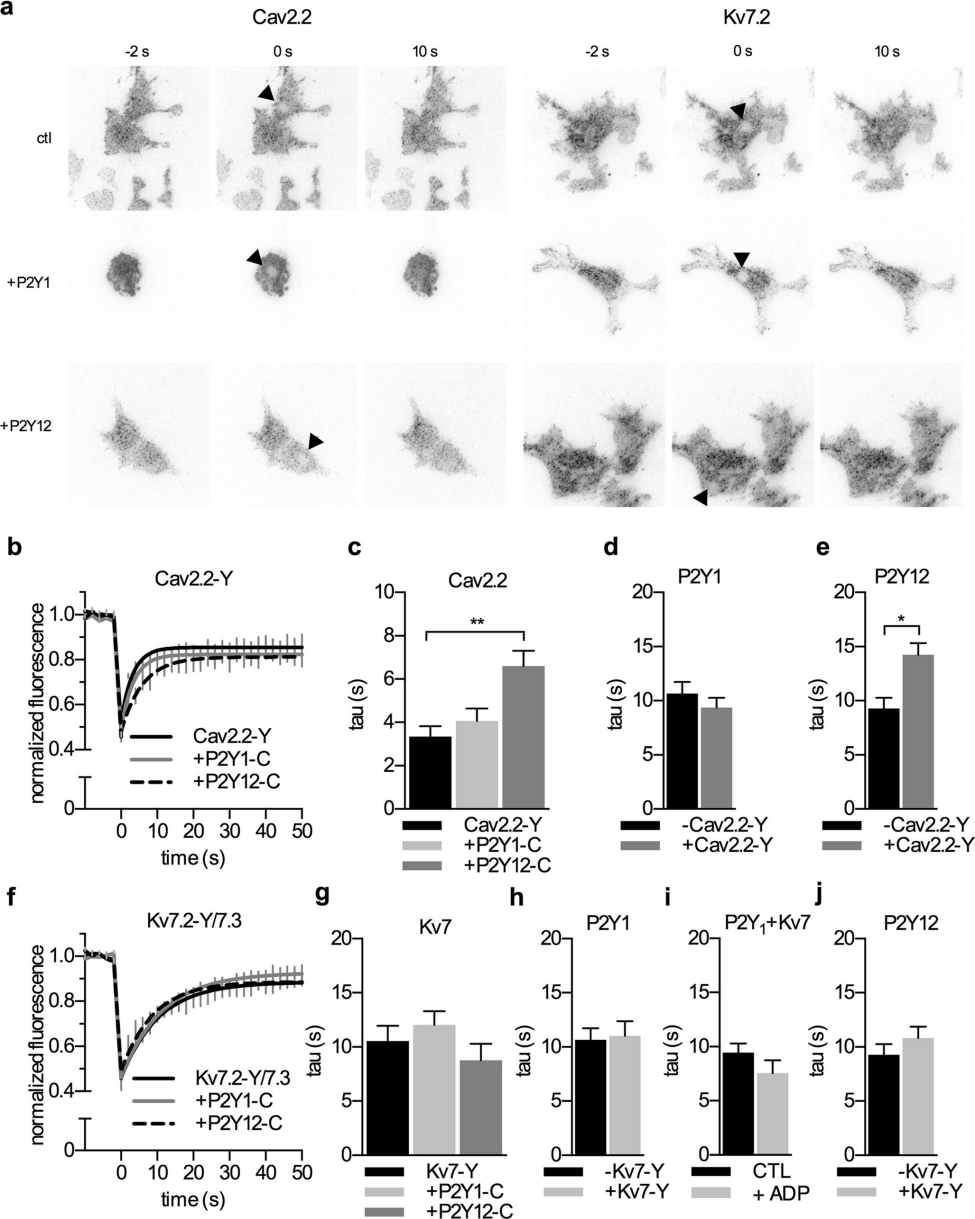
Mobility of P2Y receptors and ion channels in the membrane determined by FRAP. **a** Representative TIRF images of tsA 201 cells expressing the indicated combinations of channels and receptors taken before (−2 s) immediately after bleaching (0 s) and after 10 s recovery time. *Black arrowheads* point towards the bleaching area. **b**, **f** Time course of normalized fluorescence intensities of YFP-tagged Cav2.2 (Cav2.2-Y; *n* = 9) and Kv7.2/7.3 (Kv7.2-Y/7.3; *n* = 11) channels, respectively, expressed either alone or together with P2Y1-CFP and P2Y12-CFP. **c**, **g** *Bars* display time constants (tau) of Cav2.2-Y and Kv7.2-Y/7.3 recovery, respectively, as derived from the traces shown in **b** and **f**. In **c**, tsA 201 cells expressed Cav2.2-YFP either alone (*black*, *n* = 9) or together with P2Y1-CFP (*light gray*, *n* = 9) and P2Y12-CFP (*middle gray*, *n* = 9), respectively. In **g**, tsA cells expressed Kv7 channels either alone (*black*, *n* = 11) or together with P2Y1-C (*light gray*, *n* = 10) and P2Y12-C (*middle gray*, *n* = 11), respectively. **d**, **h** *Bars* display time constants (tau) of P2Y1-C recovery. The receptor was expressed either alone (*black*, *n* = 13) or together with Cav2.2-Y (*middle gray*, *n* = 11) and Kv7.2-Y/7.3 (*light gray*, *n* = 13), respectively. **i** *Bars* display the time constants of P2Y1-C recovery in the presence of Kv7 in absence (*black*) or presence (*gray*) of 10 μM ADP (**e**, **j**). *Bars* display time constants (tau) of P2Y12-C recovery. The receptor was expressed either alone (*black*, *n* = 11) or together with Cav2.2-Y (*middle gray*, *n* = 13) and Kv7.2-Y/7.3 (*light gray*, *n* = 10), respectively. *Asterisk* (*), *double asterisks* (**) indicate *p* < 0.05 and *p* < 0.01, respectively

To reveal whether the P2Y receptors, in addition to being in juxtaposition, might get in some physical contact with the ion channels, FRAP assays were performed. In this experimental paradigm, the mobility of ion channels can be altered by interactions with other membrane proteins such as accessory subunits [40, 41]. To focus on potential interactions within the membrane only, these experiments were performed in TIRF microscopy again. After bleaching a spot of approximately 4 μm in diameter, the recovery of fluorescence within this area was determined (Fig. 4a). The shortest recovery times were found for Cav2.2-EYFP, slower recovery was observed for Kv7.2/7.3-EYFP channels and for ECFP-tagged P2Y receptors. The extent of recovery was incomplete for all proteins, thus alluding to some immobile fractions.

When fluorescent channel proteins were coexpressed with either P2Y1 or P2Y12, this maximum of recovery remained unaltered. For Kv7.2-YFP/7.3, the rates of recovery were also the same whether the P2Y receptors were present or not (Fig. 4f, g). In contrast, the speed of recovery of Cav2.2-YFP was affected by coexpressed receptors: recovery was significantly slowed in the presence of P2Y12, but was not influenced by P2Y1 receptors (Fig. 4). Likewise, the extent of recovery observed for CFP-tagged P2Y receptors was the same whether channel proteins were present or not. The speed of recovery was only altered when P2Y12-CFP was coexpressed with Cav2.2-YFP, but not in any of the other combinations. Fluorescent recovery of P2Y1 receptors coexpressed with Kv7 heteromers was also unaffected by ADP (Fig. 4). Taken together, evidence for physical interactions was only obtained for P2Y12 and Cav2.2, but not for any other channel/receptor pair.

## Discussion

Almost all P2Y receptor subtypes have been found to control the gating of various neuronal ion channels, whether they were endogenously or heterologously expressed in different types of neuron. The ion channels that have been reported to be modulated by the largest number of different P2Y receptors are Kv7 and Cav2.2 channels [3]. Here, the regulation of these two neuronal channels via P2Y1 and/or P2Y12 receptors has been reconstituted by the transfection of tsA 201 cells. Therein, currents through these channels were not affected by ADP, unless either of these two receptors had been coexpressed with the channels.

Previously, endogenous P2Y1 receptors of rat superior cervical ganglion neurons have been shown to control the gating of Kv7 [42] and Ca^2+^ channels [23], while P2Y12 receptors mediate an inhibition of the Ca^2+^ channels only [43]. Likewise, in sensory neurons, native P2Y1 receptors were found to mediate an inhibition of both, Kv7 [44] and Ca^2+^ channels [45] channels, whereas activation of P2Y12 receptors led to the diminution of solely Ca^2+^ currents [44]. The present results precisely recapitulate these results with recombinant receptors and Kv7.2/7.3 or Cav2.2 coexpressed in tsA 201 cells: while P2Y1 receptors controlled both, currents through Kv7 and Cav2.2 channels, P2Y12 receptors only did so for Cav2.2.

In neurons, the inhibition of channels of the Cav2 family via P2Y receptors can involve two pathways: (i) activation pertussis toxin-sensitive G proteins and subsequent binding of βγ subunits to the channel proteins; (ii) depletion of membrane phosphatidylinositol 4,5-bisphosphate via phospholipase C [3]. The very same mechanisms were active in the recombinant system of tsA 201 cells. The inhibition of Ca^2+^ currents through P2Y12 receptors included a slowing of activation kinetics that is characteristic of the voltage-dependent interaction between G protein βγ subunits and channel proteins and was attenuated by pertussis toxin, but not by a phospholipase C inhibitor. Vice versa, Ca^2+^ current reduction via P2Y1 was pertussis toxin-insensitive and blunted by a phospholipase C inhibitor. Likewise, the closure of Kv7 channels via P2Y1 was abolished by the phospholipase C inhibitor and most probably involves depletion of phosphatidylinositol 4,5-bisphosphate [28]. Hence, the present results obtained in tsA 201 cells are in substantial agreement with previous data derived from neurons.

To reveal whether a certain spatial arrangement of receptors and channels might parallel the functional data reported above, various microscopic techniques were employed. P2Y1 receptors showed robust FRET signals with Kv7 and Cav2.2 in both epifluorescence widefield as well as TIRF microscopy. While *N*_FRET_ values derived from epifluorescence images can only be used for comparing the efficiency of fluorescence energy transfer between pairs of chosen FRET partners [33], the DRAP method allows for approximation of distances between FRET donors and acceptors. Assuming a Förster radius of 4.9 nm for the FRET pair used, the donor fluorescence recovery of 10 % found for P2Y1 and Kv7 channels would translate into a distance of roughly 7 nm. In contrast, the respective value obtained with P2Y12 receptors reflects at least 10 nm if not much more, as the calculation is based on the sixth power of the distance between the fluorophores relative to the Förster distance. With Cav2.2 channels, both receptors produced *N*_FRET_ values comparable to those of positive controls, and the distances calculated from the TIRF-DRAP measurements were similar and amounted to 7 nm (P2Y1) and 6.8 nm (P2Y12). These differences in distance go in parallel with the finding that P2Y1 receptors mediated inhibition of both types of channel, whereas P2Y12 receptors only did so with Cav2.2. This confirms previous reports demonstrating that GPCRs are unable to control the gating of ion channels over micrometer distances [19, 20].

In addition to analyzing the juxtaposition of P2Y receptors and ion channels via FRET, FRAP experiments were carried out in order to gain insight into potential physical interactions. The FRAP recovery time constants obtained with Kv7.2/7.3 channels were in good agreement with those of the closely related Kv7.1 channels whose mobility can be altered by the presence of KCNE subunits [40, 46]. In this study, coexpression of P2Y1 with either Cav2.2 or Kv7 did not lead to any significant alteration in FRAP recovery of either the channels or the receptor. Considering the FRET results, this might be surprising at first sight, since the complex of two proteins should show a reduced diffusion coefficient (*D*) and thus a slower recovery from bleaching. There might be several reasons to account for this apparent discrepancy. One comes from the fact that it is known that the scaling of *D* with size (*R*) is best described by an ln(1/*R*) relationship [47]. This means that a doubling in size alone would lead to a decrease of only 10–15 % in *D*, which lies within the experimental variability and thus might not be detected. An alternative explanation would be offered by dynamic transient interactions between P2Y1 and the effector channels that could not suffice to form a co-diffusing complex.

In contrast to the results obtained with P2Y1, coexpression of P2Y12 with Cav2.2 led to pronounced changes in the FRAP recovery kinetics. The more than 50 % increase in recovery time constant might be attributed to the formation of large complexes. A candidate protein potentially participating in such a larger complexes would be, for instance, NHERF1, which is known to interact with P2Y12 receptors [26, 48]. NHERF2 has been shown to be present in HEK293 cells [23], but information on the endogenous expression of NHERF1 is lacking. Other possibilities would be some kind of anchoring or partitioning into membrane microdomains with differing diffusion properties. Since anchoring would lead to a significant change in the immobile fraction of either channels or receptors, this alternative can be ruled out as the maximum of FRAP recovery was always the same. Therefore, partitioning into specialized membrane microdomains needs to be considered. Indeed, P2Y12 receptors have been shown repeatedly to be present in lipid rafts [49]. In platelets, for instance, signaling via P2Y12, but not via P2Y1, requires intact lipid rafts, as it was inhibited by methyl-β-cyclodextrin [50]. Likewise, in non-neuronal cells, a considerable proportion of heterologously expressed Cav2.2 channels can be found within lipid rafts [50, 51]. In contrast, Kv7 channels as well as regulating receptors may reside inside [52] as well as outside of lipid rafts [53]. Therefore, the finding that P2Y12 receptors affected the mobility of Cav2.2 channels and vice versa may be explained by a redistribution of these proteins between lipid rafts and non-raft compartments of the plasma membrane that depends on the presence of the respective other one. Along the same line, the apparent lack of physical interaction between P2Y1 receptors and Kv7 channels as observed in FRAP experiments may be due to the fact that both proteins are equally distributed within the same membrane compartments. Alternatively, weak interactions between these two may suffice to lead to juxtaposition and physiologically relevant cross talk, but escape detection as assayed by FRAP recovery. Obviously, two membrane proteins that are not juxtaposed to each other, such as P2Y12 receptors and Kv7 channels, cannot influence each other’s mobility.

The present results regarding juxtaposition of and physical interactions between P2Y receptors and ion channels appear to be related to the interposed signaling cascades. Physical interaction was only detected between an ion channel and a GPCR which are functionally connected by the direct interaction of G protein βγ subunits with the pore forming protein (i.e., Cav2.2 and P2Y12). Previously, juxtaposition has been reported for Kir3.1/3.2 and activating GPCRs [30, 31] which also regulate channel gating via direct G protein βγ interactions. Moreover, GABAB and Kir3 channels are both known to associate with lipid rafts GPCRs [30] as mentioned for P2Y12 and Cav2.2 channels above. Hence, lipid rafts may provide optimal conditions for ion channel regulation via direct βγ interactions, but additional experimental evidence is required to further substantiate this notion.

In summary, the ADP receptors P2Y1 and P2Y12 control the gating of Kv7 and Cav2.2 channels in a selective manner, and the regulation of each of these neuronal ion channels is paralleled by juxtaposition of channel and P2Y receptor proteins within appropriate membrane microdomains.
